# Joy in Long Term Services and Supports Caregiving

**DOI:** 10.1093/geroni/igaf122.631

**Published:** 2025-12-31

**Authors:** Nathan Boucher, Lillian Hung

## Abstract

Retaining and empowering staff in long-term services and supports (LTSS) is essential for sustaining quality care. Despite high demands, staff often receive low wages and limited recognition, making workplace culture, professional growth, and well-being critical factors in job satisfaction/retention. This symposium presents findings from three qualitative studies examining staff experiences in LTSS, highlighting factors fostering retention, resilience, and joy in caregiving. The first study explored retention factors among Canadian LTSS staff through focus groups, identifying three key themes: (1) professional growth and development through training and mentorship, (2) recognition and workplace culture that foster team cohesion and appreciation, and (3) relational joy in caregiving, where meaningful connections with residents and colleagues enhanced resilience. The second study analyzed essays from LTSS clinicians reflecting on moments of joy in their work. Content analysis revealed that joy stemmed from (1) building relationships through residents’ unique life stories, (2) teamwork that fostered belonging, and (3) shared decision-making that supported person-centered, end-of-life care. Despite workforce shortages, clinicians found fulfillment through narrative medicine, celebrating direct care staff, and engaging in palliative care discussions. The third study examined experiences of LTSS administrators during the COVID-19 pandemic. Findings underscored the importance of communication, collaboration, and relational leadership in sustaining staff morale. Innovative communication strategies helped alleviate isolation and reinforced a sense of team belonging. Collectively, these studies in Canada and the US underscore the need for evidence-based retention strategies that prioritize professional development, workplace recognition, and relational well-being to sustain and strengthen the joy of the LTSS workforce.

